# Case Report: Chemotherapy-induced phlebitis in a dog: diagnostic approach and management strategies

**DOI:** 10.3389/fvets.2025.1628931

**Published:** 2025-10-06

**Authors:** Seoyoung Hwang, Heejeong Hong, Joohyun Jung

**Affiliations:** Ilsan Animal Medical Center, Goyang, Republic of Korea

**Keywords:** chemotherapy-induced phlebitis, doxorubicin, carboplatin, ultrasonography, low-level laser therapy

## Abstract

A 7-year-old neutered male Golden Retriever diagnosed with osteosarcoma of the left radius underwent limb amputation followed by adjuvant chemotherapy utilizing alternating carboplatin-doxorubicin protocols. Following doxorubicin administration, the patient exhibited localized heat, swelling, pain, and lameness in the right forelimb, indicative of phlebitis. Ultrasonography confirmed chemotherapy-induced phlebitis with thickening of the right cephalic vein wall, intraluminal thrombi, and significant perivascular edema. Treatment involved anti-inflammatory corticosteroids, oral medications (clopidogrel, pentoxifylline, doxycycline, vitamin E), and low-level laser therapy (LLLT), achieving clinical improvement. Despite successful control of chronic inflammation in the cephalic vein, phlebitis subsequently developed in other veins, including the right common dorsal digital vein and left saphenous vein, despite varying vascular access points. The patient showed clinical improvement with the combined use of oral medication and low-level laser therapy. The chemotherapy regimen was completed successfully without osteosarcoma recurrence, and the patient remained stable for over 13 months post-treatment. Following discontinuation of chemotherapy and supportive care, no further progression of phlebitis occurred. To the authors' knowledge, this report represents the first documented veterinary case of chemotherapy-induced phlebitis in a dog. While extensively reported in human oncology, chemotherapy-induced phlebitis remains underreported in veterinary medicine. Clinicians should recognize phlebitis as a potential complication associated with chemotherapeutic agents such as doxorubicin and carboplatin. Ultrasonography serves as an essential diagnostic and monitoring tool. Prophylactic corticosteroids and adjunctive low-level laser therapy offer promising preventative and therapeutic strategies, particularly for patients with predisposed vascular inflammation. This case underscores the importance of early identification, proactive management, and individualized treatment approaches to chemotherapy-associated phlebitis in veterinary oncology.

## Introduction

Phlebitis, an inflammatory condition of the veins, frequently occurs as a complication in patients undergoing intravenous catheterization. Clinically, it is characterized by symptoms such as erythema, pain, swelling, and localized warmth (1). In human medicine, catheter-associated phlebitis has been extensively studied, with an incidence rate of approximately 31.4% reported in patients receiving intravenous infusions (2). Similarly, phlebitis is commonly observed in dogs and can typically be attributed to chemical, infectious, or mechanical irritation (3). In humans, chemical phlebitis often results from endothelial damage induced by medications, influenced by factors such as drug pH and osmolarity (4). Numerous studies have documented that hypertonic solutions, vasoactive medications, antibiotics, and chemotherapeutic agents are associated with phlebitis in humans. Notably, about 70% of cancer patients undergoing chemotherapy experience phlebitis as a side effect (5). Chemotherapeutic agents implicated in causing phlebitis include platinum compounds, cyclophosphamide, and 5-fluorouracil, prompting ongoing research into preventive and management strategies (6). However, comprehensive documentation of chemical phlebitis-inducing medications in dogs remains limited. Existing veterinary research predominantly focuses on mechanical causes of phlebitis and the relationship between the duration of intravenous catheter placement and phlebitis development (7–12). This study aims to investigate chemotherapy-induced phlebitis and discuss subsequent management strategies, addressing a gap previously unexplored in veterinary literature.

## Case description

**Figure 1:**
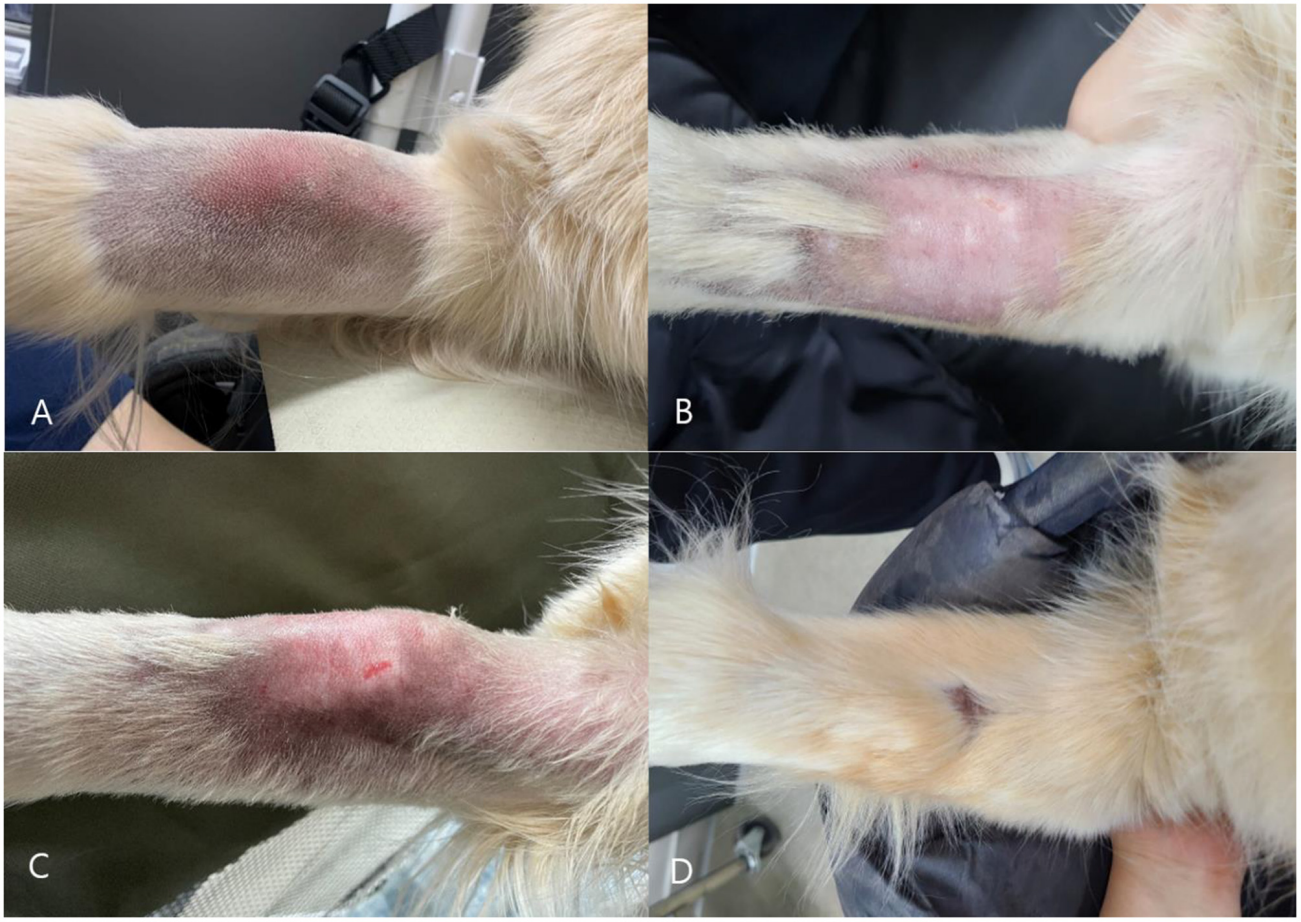
**(A)** Gross lesion on the patient's right forelimb at the onset of phlebitis, accompanied by swelling, heat, and significant pain. **(B)** Improvement of the gross lesion on the patient's right forelimb after management of phlebitis, with resolution of swelling, heat, and pain. **(C)** Worsening of the gross lesion on the right forelimb following the second carboplatin infusion via the common dorsal digital vein of the right hindlimb; the lesion subsequently improved after short-term administration of low-dose steroids. **(D)** Normal hair regrowth following treatment of phlebitis.

**Figure 2:**
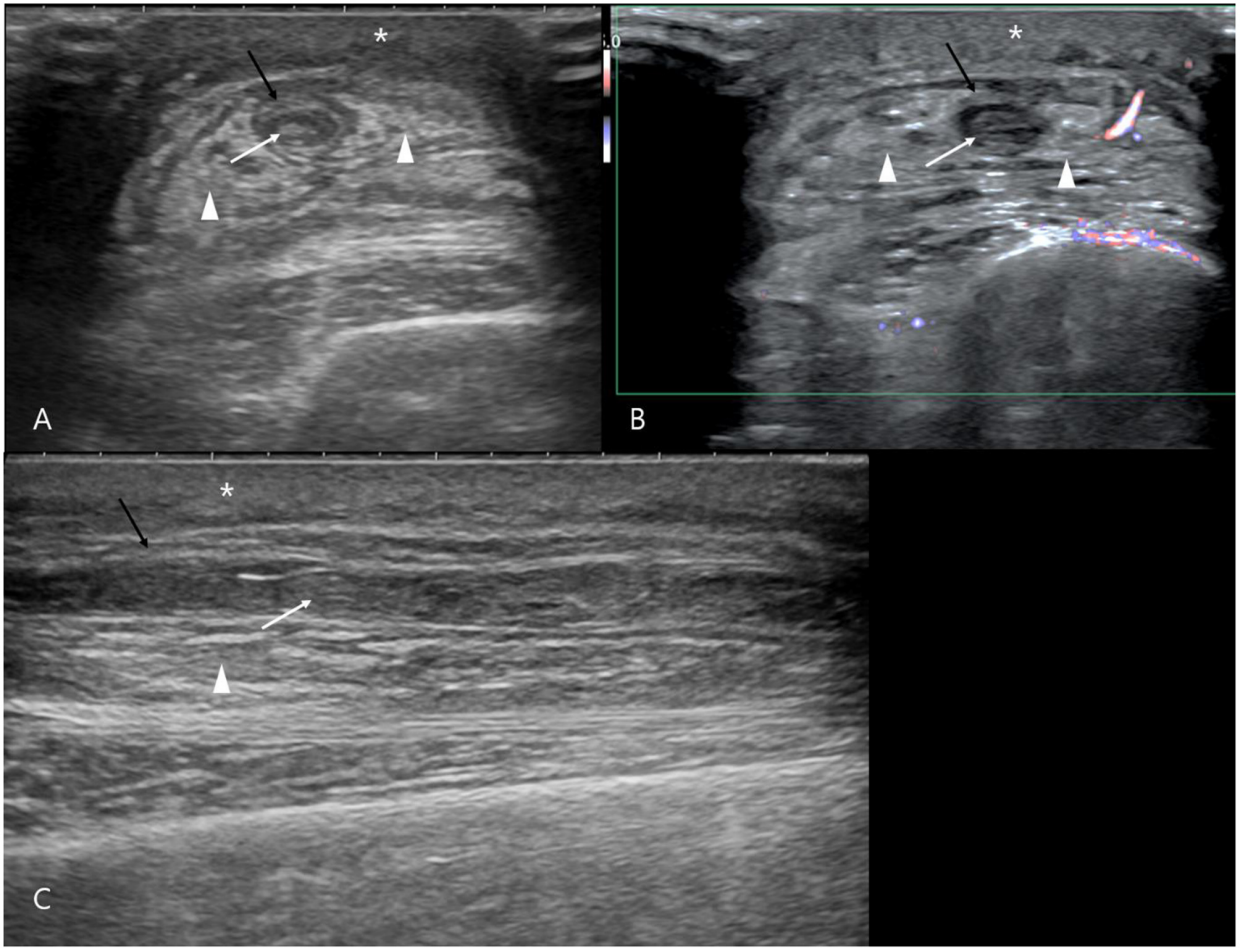
Ultrasonographic findings of chemotherapy-induced phlebitis in the right cephalic vein. **(A)** Transverse view, **(B)** Color Doppler imaging, **(C)** Sagittal view. Severe subcutaneous edema beneath the skin (asterisks), fat edema surrounding the right cephalic vein (arrowheads), irregular thickening of the vein wall (black arrows), and heterogeneous hyperechoic intraluminal thrombi are observed (white arrows).

A 7-year-old, neutered male Golden Retriever was diagnosed with osteosarcoma of the left radius at another hospital and was referred to Ilsan Animal Medical Center for further treatment after amputation. Although there was no suspicion of distant or near metastasis on the CT scan performed before surgery, it was decided to proceed with adjuvant chemotherapy. Carboplatin-doxorubicin alternating protocol for a total of 3 cycles, q21d, with doses of carboplatin 250 mg/m^2^ IV and doxorubicin 25 mg/m^2^ IV, was adopted. The first chemotherapy was administered through the right cephalic vein. There were no remarkable findings before or after the carboplatin injection, and the patient's clinical symptoms after chemotherapy were also good. Three weeks later, doxorubicin chemotherapy was also administered through the right cephalic vein. There were no unusual events during the procedure, but just before the end of chemotherapy, pressure was applied to the syringe piston, making it impossible to inject a small amount of doxorubicin. Because the outflow of blood through the catheter was extremely good, it was considered that the catheter was properly positioned within the blood vessel, so extravasation was not suspected, but the drug could not be administered, so about 2.2 mg/m^2^ of doxorubicin could not be injected. On the next visit, the owner said that the patient had edema in the right forelimb for about 3 days after chemotherapy, but it had subsided well. There were no unusual findings during the in-hospital physical examination. However, on the 18th day after chemotherapy, the patient visited the hospital because of lameness, pain, and swelling in the right forelimb that suddenly occurred that day. It was accompanied by depression and anorexia. On physical examination, the body temperature was 39.7 °C, and there was significant heat, redness, and swelling around the right cephalic vein where chemotherapy was performed (Figure 1A). During blood tests, complete blood count, serum chemistry, venous blood gas analysis, and C-reactive protein were all unremarkable, and d-dimer was also within the normal range. When the right cephalic vein was evaluated by ultrasound, an irregular and thickened wall of the right cephalic vein was observed, along with numerous thrombi inside, and significant edema of the surrounding soft tissue (Figure 2). Distal blood flow in the right cephalic vein was not confirmed. Taking all of this together, the patient was diagnosed as having phlebitis in the right cephalic vein. The chemotherapy schedule was halted, and anti-inflammatory corticosteroids were administered to manage phlebitis, along with low-level laser therapy (LLLT). Corticosteroid therapy was initiated at 0.5 mg/kg PO BID, but due to severe polyuria, polydipsia, and panting that markedly interfered with daily activities, the dose was tapered to 0.3 mg/kg PO SID, which was sufficient to control limb swelling without adverse effect. The corticosteroids were discontinued once the phlebitis-related clinical signs (lameness, swelling, heat, and pain) had resolved and the limb condition had stabilized, leaving only chronic skin changes such as thickening and localized alopecia at the lesion site. Additional medications included pentoxifylline (10 mg/kg PO BID), clopidogrel (2 mg/kg PO SID), vitamin E (400 IU/dose PO BID), and doxycycline (5 mg/kg PO BID). LLLT was performed three times per week, specifically using an MLS^®^ laser device (M6-ASA; Arcugnano, Italy), which represents a specific form of LLLT utilizing synchronized dual wavelengths (808 nm continuous and 905 nm pulsed). The arthrosis preset (phase 2) was applied with a frequency of 292 Hz, a mean power of 1.5 W, an energy density of 4.04 J/cm^2^, and a total energy delivery of approximately 605 J over a 19-min session, targeting a spot area of 150 cm^2^. Clinical symptoms such as depression, anorexia, lameness, and edema were all improved following drug prescription and LLLT. However, the phlebitis found on ultrasound improved to some extent but did not completely disappear. The thickening, edema, and inflammation of the right cephalic vein and surrounding soft tissues continued, and it was determined that the inflammation had become chronic. After stabilizing the phlebitis of the right cephalic vein to some extent (Figure 1B), chemotherapy was restarted after 72 days. It was decided to exclude doxorubicin, which caused severe phlebitis, from the protocol, and to continue the chemotherapy schedule with only carboplatin. Second and third carboplatin were injected through the common dorsal digital vein of the right hindlimb. The phlebitis of the right cephalic vein worsened after second injection of carboplatin (Figure 1C), but improved after taking low-dose steroids for a short period of time. During the third injection of carboplatin, steroids were prescribed preemptively. A week after the chemotherapy, there were no events in the right forelimb, but edema appeared near the injection site of the right hindlimb, and phlebitis was confirmed in the right common dorsal digital vein on ultrasound. LLLT was also performed on this lesion, and oral medications for phlebitis (pentoxifylline, clopidogrel, vitamin E, doxycycline, and short-term corticosteroid immediately after chemotherapy) were consistently prescribed. Afterwards, the patient took a break from chemotherapy to manage phlebitis and undergo surgery and recovery for an incidental low-grade dermal mast cell tumor, which was unrelated to the osteosarcoma. The fourth carboplatin injection was performed approximately 2 months after the third carboplatin injection. It was injected through the left saphenous vein, and steroids were prescribed preemptively. Two weeks later, the patient visited the clinic. There were no clinical symptoms in all three legs, but phlebitis was confirmed in the left saphenous vein on ultrasound. This patient's intravenous chemotherapy was completed and he switched to oral chemotherapy (cyclophosphamide at 219 mg/m^2^, administered orally once every 3 weeks) according to the will of his owner. A total 14 cycles of cyclophosphamide were administered, each given concurrently with oral furosemide (20 mg/dose, approximately 0.55 mg/kg). Midway through the course, the protocol was transitioned to toceranib (2.75 mg/kg, three times per week) for 3 months due to concerns about potential long-term adverse effects of cyclophosphamide, including hemorrhagic cystitis and myelosuppression. However, cyclophosphamide was resumed at the owner's request, prompted by financial constraints. The patient is currently under post-chemotherapy surveillance and, 2 years after surgery, remains in good condition with no evidence of local recurrence or distant metastasis of osteosarcoma. Phlebitis management was continued by consistently administering oral medications including clopidogrel, pentoxifylline, vitamin E, and doxycycline and LLLT for all lesions. The phlebitis of the right cephalic vein became chronic and showed no further improvement, but the phlebitis of the right common dorsal digital vein and left saphenous vein waxed and waned, and in particular, the phlebitis of the right common dorsal digital vein recovered well. The progression of phlebitis was assessed comprehensively based on clinical signs, gross lesion, and ultrasonographic findings. Since phlebitis and clinical symptoms were judged to be good, the patient stopped taking oral medication and LLLT about 8 months after the first phlebitis. The patient is being monitored, and is doing well without any further progression. Currently, this patient is surviving well for 13 months after amputation of the left forelimb because of osteosarcoma, without recurrence. Phlebitis that occurred at three injection sites after five rounds of chemotherapy has also remained stable (Figure 1D).

## Discussion

In veterinary medicine, peripheral intravenous catheterization is one of the most frequently employed methods for administering medications and fluids. Phlebitis, a commonly observed complication in hospitalized dogs, predominantly results from physical irritation or infections associated with catheter placement (7, 13, 14). Unlike human medicine, however, chemical-induced phlebitis resulting from drug administration is rarely documented in veterinary literature. To the authors' knowledge, this report describes the first veterinary case of chemically induced phlebitis. In human medicine, several medications, including chemotherapeutic agents such as doxorubicin and carboplatin, have been associated with phlebitis (6, 15, 16). Consequently, veterinarians should remain vigilant regarding the potential for drug-induced phlebitis when administering intravenous medications documented as irritants in human medicine, implementing appropriate preventative and therapeutic measures when necessary. Ultrasonography is valuable for diagnosing phlebitis in veterinary patients. Typical ultrasonographic features of phlebitis include irregular thickening of the affected vein walls, intraluminal thrombi with heterogeneous echogenicity, diminished or absent vascular blood flow, and marked edema of adjacent soft tissues. In the current case, the ultrasonographic findings were most severe in the right cephalic vein, with similar lesions also detected in the right common dorsal digital vein and the left saphenous vein. These findings align with previously reported canine vascular ultrasonographic characteristics of phlebitis (3). In this patient, diagnosis was based on clinical signs such as localized heat, inflammation, pain, lameness, and confirmatory ultrasound findings. Notably, systemic inflammation markers and coagulation parameters remained normal, highlighting ultrasonography's diagnostic and monitoring utility. In human medicine, ultrasound examination is similarly integral to phlebitis diagnosis (17). Therefore, ultrasonographic evaluation is recommended when clinical suspicion arises. Although chemical phlebitis has not been previously reported in veterinary medicine, this case suggests that certain patients may experience recurrent episodes following exposure to specific drugs. For patients with a prior history of chemical-induced phlebitis, clinicians should exercise caution when administering additional potentially irritating drugs and consider proactive ultrasound evaluation at injection sites, even without overt clinical symptoms, enabling early intervention and optimal outcomes. In the present case, difficulty administering doxorubicin was attributed to vessel inflammation and edema, suggesting an underlying initial phlebitis possibly induced after the first carboplatin administration. Severe phlebitis subsequently developed following doxorubicin administration at the same site. Therefore, clinical signs indicative of phlebitis should prompt immediate chemotherapy cessation and further diagnostic evaluation, aiming to reduce patient discomfort and prevent complications. Early detection and proactive management significantly influence overall therapeutic success. In this case, the phlebitis in the right cephalic vein recurred and worsened after drug administration through other limbs. This may be explained by the risk of exacerbation of existing phlebitis lesions when additional chemical stimulants are administered, even via different vessels. Due to systemic circulation, inflammatory mediators may intensify at sites with pre-existing phlebitis. Consequently, patients with existing phlebitis require careful consideration and close monitoring during subsequent drug administrations, particularly during ongoing chemotherapy protocols. Steroids are recognized for their analgesic and anti-inflammatory properties, providing effective relief in phlebitis management (18). Although the prophylactic use of steroids remains debated due to potential systemic side effects, in this patient, preemptive administration significantly prevented or minimized phlebitis-related clinical symptoms. Therefore, individualized steroid therapy, tailored according to the patient's underlying conditions and overall health status, can play a pivotal role in preventing treatment-related complications and improving clinical outcomes. In managing phlebitis in this patient, pentoxifylline, doxycycline, and vitamin E were included in addition to corticosteroids (19). Pentoxifylline is commonly used in canine cutaneous vasculitis due to its ability to improve microcirculation and reduce inflammation. Doxycycline is widely utilized for its anti-inflammatory and immunomodulatory effects in vascular skin disorders (20), and vitamin E provides antioxidant support to promote vascular healing (21). Although controlled clinical trials specific to canine phlebitis are limited, these agents are extensively used in practice to support vessel integrity and mitigate inflammation. Low-level laser therapy (LLLT) is known for its anti-inflammatory properties and has demonstrated effectiveness in human phlebitis management (22). In the present case, LLLT was initiated concurrently with medical treatment, and the patient showed clinical improvement during combined therapy. While veterinary reports on LLLT for phlebitis management are currently lacking, its convenience, affordability, and minimal patient stress support its broader application in veterinary practice. Furthermore, evidence from human phlebitis and canine inflammatory disorders highlights its anti-inflammatory potential (22–24). Combined with the favorable outcome in this case, these findings warrant further investigation and controlled trials to clarify the role of LLLT as adjunct therapy for veterinary phlebitis. Because LLLT was applied in combination with medical therapy, its individual contribution remains unclear, and further controlled studies are warranted to elucidate its independent efficacy in veterinary phlebitis. Additional therapeutic approaches from human medicine include topical steroid ointments (25) and sesame oil applications (26) to manage chemotherapy-induced phlebitis. Though not employed in this case, these treatments may present viable options for veterinary patients given their localized administration and minimal systemic adverse effects, warranting further veterinary research. In conclusion, this report documents the first veterinary case of chemical-induced phlebitis, highlighting parallels with human medicine regarding chemotherapy-related vascular complications. Clinical suspicion, ultrasonographic diagnosis, and vigilant monitoring are essential components of effective phlebitis management. Combined pharmacological strategies, including oral medications, steroids, and adjunctive LLLT, significantly enhance therapeutic outcomes in managing and preventing phlebitis complications during veterinary chemotherapy.

## Data Availability

The original contributions presented in the study are included in the article/supplementary material, further inquiries can be directed to the corresponding author.
